# Sensitivity analysis of closed-loop one-chamber and four-chamber models with baroreflex

**DOI:** 10.1371/journal.pcbi.1012377

**Published:** 2024-12-23

**Authors:** Karolina Tlałka, Harry Saxton, Ian Halliday, Xu Xu, Andrew Narracott, Daniel Taylor, Maciej Malawski

**Affiliations:** 1 Sano Centre for Computational Medicine, Cracow, Poland; 2 Division of Clinical Medicine, University of Sheffield, Sheffield, United Kingdom; 3 School of Computer Science, University of Sheffield, Sheffield, United Kingdom; 4 Insigneo Institute for in silico Medicine, University of Sheffield, Sheffield, United Kingdom; 5 Department of Computer Science, AGH University of Science and Technology, Cracow, Poland; University of Michigan, UNITED STATES OF AMERICA

## Abstract

The baroreflex is one of the most important control mechanisms in the human cardiovascular system. This work utilises a closed-loop in silico model of baroreflex regulation, coupled to pulsatile mechanical models with (i) one heart chamber and 36-parameters and (ii) four chambers and 51 parameters. We perform the first global sensitivity analysis of these closed-loop systems which considers *both* cardiovascular and baroreflex parameters, and compare the models with their respective unregulated equivalents. Results show the reduced influence of regulated parameters compared to unregulated equivalents and that, in the physiological resting state, model outputs (pressures, heart rate, cardiac output etc.) are most sensitive to parasympathetic arc parameters. This work provides insight into the effects of regulation and model input parameter influence on clinical metrics, and constitutes a first step to understanding the role of regulation in models for personalised healthcare.

## Author summary

In the era of personalised healthcare, there is a growing need to develop computational models that accurately represent human physiology. Here, we examine two models of the human circulatory system, incorporating the effects of baroreflex regulation— a key neural homeostatic mechanism responsible for controlling blood pressure during rest and exercise on short timescales. We investigate the impact of including baroreflex regulation on clinically significant metrics such as cardiac output. Due to the model’s complexity and a large number of parameters, quantifying the effects of regulation in a closed-loop operation was previously deemed infeasible, but by utilising an efficient in silico encapsulation and high-performance computing we are able, here, to quantify the baroreflex’s impact on mechanical outputs.

## Introduction

Society faces a number of challenges related to the need to improve quality of life in an ageing population with limited resources. Advances in healthcare are crucial, especially for the treatment of cardiovascular diseases (i.e., heart failure, valvular disease, and peripheral vascular disorders); the question is how to proceed efficiently. Personalised in silico medicine provides a strategy to optimise healthcare delivery, through improved diagnostic power and the ability to stratify patients to optimal management cohorts [1]. The main assumptions of this approach are that (i) medical therapy should be individualised, and (ii) some aspects, like effectiveness of treatment or risk of disease, can be predicted. Quite apart from improving the standard of care, a more personalised approach may also optimise the efficiency and cost-effectiveness of healthcare services. Of course, implementation faces some ethical and technical challenges, such as data sharing and the need for legislative reform [2].

One of the most important directions for the development of personalised medicine is the digital twin, in which the main objective is to create a digital representation of a patient—a mathematical model encapsulating key physiological mechanisms possessing an ability to evolve and adapt after the physical individual. Models can be, broadly, “physics-driven” i.e. based on physical laws, generally deterministic and selective about the physical and physiological mechanisms they depict, or data-driven i.e. based on statistical analysis of medical datasets and possibly stochastic [3]. We consider a cardiovascular system model compiled from a range of low-order sub-models. Such models are inherently adaptable and so vary widely in the literature in their execution time, application, spatial resolution of outputs and physiological detail [4, 5]. The most detailed data are provided by three-dimensional (3D) analysis, but these require huge computational resources and are limited in scope to device or organ scales. A lower resolution, one-dimensional (1D) approach reduces computational expense by basing a description on the cross- sectional integral of velocity in (say) an artery, with a commensurate loss of output information on hemodynamic velocity and pressure distributions. On the other hand, it is possible to incorporate certain mechanical properties of vessel walls within 1D descriptions [6] and to address the spatial scales of the arterial tree [7]. A coarse-graining in space of a 1D formulation leads to a zero-dimensional (0D) model, also known as an electrical analogue or lumped-parameter model (LPM) [4]. This technique exploits the analogy between electrical and hydraulic circuit theory and formal association of pressure and potential difference and volumetric discharge (or flow) and current, to describe a set of units representing cardiovascular tree compartments (significant vessels, or sets of vessels, etc.). A limitation of LPMs is that they cannot compute flow patterns within a cross-section or wave propagation phenomena. However, the simplifications (and computational cost reductions) embedded with LPMs allow them to describe flow and pressure in the entire cardiovascular system with a single framework.

Each compartment of a LPM is characterised by a small set of components, all with known parameters (the so-called model input parameters, or model factors) which determine the dynamics associated with that compartment. For example, a resistor and a parallel capacitor together represent the flow characteristics of a large vein, which from the hemodynamic perspective are dictated by frictional resistance and compliance [4]. Ideally, model outputs are compared with clinical measurements to calibrate or “tune” input parameters, “personalising” the model so that it agrees with observations. Examination of the resulting input parameters may then provide clinical insights into the status of the patient. Complex LPMs can often contain many input parameters so to personalise a model one must understand which of the compartmental parameters have the greatest impact on a chosen set of outputs, known to be clinically available. If an input parameter contributes strongly to an output, it is regarded as sensitive [8]. The set of sensitive model input parameters then become the prime candidates for personalisation.

Neural homeostatic mechanisms exert a significant influence over the cardiovascular system in both resting and exercise states [9]. The baroreflex is a physiological control mechanism that responds to mechanical stresses transduced from the aortic and carotid arterial walls. It regulates blood pressure by modifying the values of e.g. systemic vascular resistance and other so-called system effectors [10]. Dysfunction of the baroreflex is connected with common pathologies, including diabetes [11], respiratory sinus arrhythmia [12], orthostatic stress [13] and exercise intolerance [14]. And since there is no such thing as an unregulated physiological state, a suitable description and implementation of the baroreflex alongside a base cardiovascular mechanical model is a pivotal step—especially in personalised cardiovascular medicine development which seeks to describe blood pressure and flow, over the patient physiological envelope. One obstacle to implementing baroreflex regulation into LPMs is the parameterisation of neuronal pathways. Accordingly, an understanding of the influence of regulation parameters on clinically relevant measurements is of central importance, to inform both modelling and clinical choices [15].

### Background

Baroreflex modelling has been the subject of many studies [10, 15–19] as it influences blood pressure by short-term regulation (minutes) of heart rate, myocardial contractility and vessel properties (systemic resistance, venous unstressed volume) and so is important in much physiology and pathophysiology. Closed-loop modelling of the baroreflex requires a representation of neural pathways (using frequencies in the dimension of spikes per second) and a coupled cardiovascular system [10], but it is also possible to study regulation as a mechanism specified in terms of changes in pressure (see simple model in [20]).

Baroregulation models can be analysed in open-loop [15] and closed-loop conditions [10, 16], and they can be coupled with other physiological mechanisms, such as the vestibulo-sympathetic reflex [15]. One of the most complex models to include a baroreflex representation was the work of Guyton et al. [18], which presented circulatory control systems covering the dynamics of the capillary membrane, stress relaxation, hormonal control and local autoregulation.

**Fig 1:**
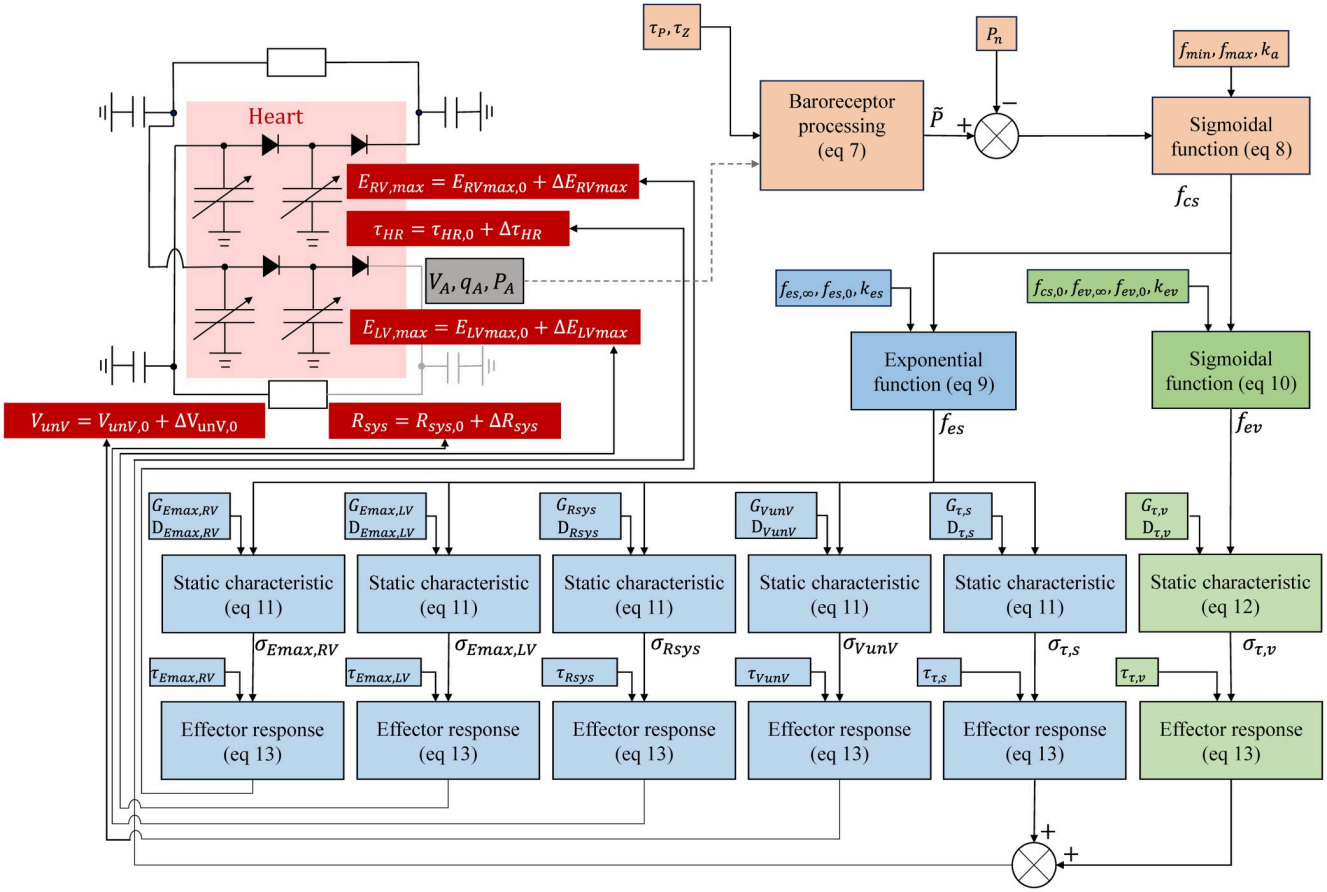
Schematic representation of information exchange between mechanical model and baroreflex representation proposed by Ursino [10], with particular emphasis on the processing within the nervous system, distinguishing afferent (orange blocks), sympathetic (blue blocks) and vagal (green blocks) components.

An extensive physiological description of the baroreflex mechanism is presented by Armstrong [21]. The mechanical stretch, sensed by baroreceptors in the carotid sinus and aortic arch, are converted into nervous action potentials and carried via afferent nerves to the brain. Here, they are processed, and an appropriate effector response is generated, which is transmitted via efferent nerves (sympathetic and parasympathetic) to target tissues (capillary bed, heart, etc.), which effect pressure changes in the cardiovascular system [21]. A schematic representation of our baroreflex model is presented in Fig 1. Heart rate and heart contractility are controlled “beat-to-beat” (i.e. variable but constant over a particular heart cycle) while systemic resistance and venous properties are adjusted continuously [22].

The mathematical description of the baroreflex, proposed by Ursino [10], is based on experiments on dogs (see [10] for the detailed references), and addresses the principal aspects of neural processing as follows. (i) the afferent processing of baroreceptors is represented by a sigmoidal shape function; (iia) the efferent sympathetic processing follows an exponential decay (iib) the efferent parasympathetic (vagal) signals follows a sigmoidal shape; (iii) delays are included with the parasympathetic response (delay 0.2 s) faster than the sympathetic (delay 2 s) the longest delay being that in unstressed volume regulation (5 s) [10, 23]. An alternative approach, including the effect of neurotransmitters, omitted by Ursino, focused on heart rate regulation, was presented by Olufsen et al. [15]. Here the authors connected four submodels (effectively block diagrams) of the baroreflex- an afferent block, a central nervous system block, a neurotransmission block and an effector model. Neurotransmitters notwithstanding, a complete description, sound physiological basis, mathematical consistency and relative simplicity motivates our choice of Ursino’s baroreflex representation in this work [10]. Other models of the functionality of baroreceptor transmitters have been proposed; however, Ursino’s remains among the most popular. For further information see Ottesen and Olufsen [24] and the references therein.

Owing to the large number of parameters and the long computation times, the prior art leans towards non-pulsatile regulated models. Hernandez et al. [25] performed a Morris-based global sensitivity analysis on a modified version of the Guyton model. Calvo et al. [26], performed a Sobol analysis on a non-pulsatile model, applied to a head up tilt test, to quantity the effects of baroregulation and mechanical parameters on heart rate and systolic blood pressure. Calvo highlighted the relevant influence of the intrinsic heart rate and the sympathetic and parasympathetic baroreflex gains on heart rate regulation, as well as the impact of left ventricle diastolic parameters on systolic blood pressure. Similar methodologies have been applied to investigate different physical processes, such as brain stem adaptation and slow breathing [27, 28]. Complimenting sensitivity analysis for model understanding, open-loop modelling of baroregulation and sensitivity analysis has been combined to obtain a subset of personalisable input parameters which can be calibrated to data. Ottesen et al. [29] used an open-loop model of heart rate regulation with the structural correlations method (see below) to reduce the input parameter space dimension to one which can more easily describe experimentally observed data. A similar approach was applied by Mahdi et al. [30], who modelled the afferent dynamics of baroregulation. The latter also used the structural correlations method to compare which model was most suitable for calibration to experimental data. The structural correlations method was initially developed by Olufsen and Ottesen [31] and applied to a model of heart rate regulation. The work they highlighted how complex models of baroregulation cannot possibly be identifiable; thus one may reduce the input set to one which is. Closed-loop models also have a history [10, 32]. Ursino [10] performed a one-at-the-time study of a closed-loop model by individually setting the strength of each regulation mechanism to zero, directing the study only to the influence of baroreflex effectors. Gee et al. [32] performed more detailed global analysis by investigating the role of particular regulation path parameters, but did not investigate the role of mechanical cardiovascular parameters in shaping overall model response.

### Study justification

When creating personalisable cardiovascular models, it is important to understand which physical processes—mechanical or neurological—need to be represented. Sensitivity analysis is the canonical first step in determining a cascade of importance. To the authors’ knowledge, no sensitivity analysis of this physical process in a pulsatile model, examining both baroreflex and mechanical parameters, has been conducted. By coupling the baroreflex regulation to a one- and four-chamber LPM, we investigate the influence of regulation parameters on clinically important model outputs as a precursor to model reduction and eventual personalisation. Previous work from our group [23] undertook a local sensitivity analysis, computed using a finite difference method, of a one-chamber closed-loop *in-silico* model of the human baro-regulation., where we compare parameter sensitivity for an unregulated and regulated one-chamber model with parameters varied ±5% and ±10% from base state. Building on this initial work we here utilise an extend heart model from which global and local sensitivity analyses are performed. We also implemented a more efficient representation of regulation, eliminating the need for global variables and migrating from a Matlab implementation using output functions [23] to and open source implementation (Julia DDE solver; algorithm Tsit5() [33, 34]). This change of model implementation, fully described in section *Model Implementation*, allows us to perform the first global analysis on a model of this class. Collectively, the essential contributions of the present work are:

1. **Impact of baroreflex regulation:** We compare the first sensitivity results of a pulsatile one- and four- chamber model with or without regulation.
2. **Level of model complexity**: We address the perennial question of the level of complexity needed when creating the cardiovascular models and the impact this has on parameter interpretation.
3. **Model implementation:** We propose an implementation of blood volume and neuronal regulation which preserves a computationally efficient delay differential equation (DDE) system formulation which can be leveraged in a global sensitivity analysis.

## Materials and methods

### Models

Two mechanical models were implemented—a simple one-chamber closed-loop representation, and a more complex four-chamber model with pulmonary and systemic loops. See sections *Mechanical One-Chamber Model* and *Mechanical Four-Chamber Model* respectively. To regulate the mechanical models, they are connected to the baroreflex model defined in section *Baroreflex Regulation*. Mathematically, our closed system is conveniently expressed in the state-space form

$$\begin{array}{c} \frac{d}{dt}\underline{X}\left(t\right)=\underline{f}(\underline{X}\left(t\right);\underline{\theta }),\;\underline{Y}\left(t\right)=\underline{h}\left(\underline{X}\left(t\right)\right), \end{array}$$ (1)

in which $\underline{\theta }$ denotes an input parameter vector, $\underline{X}$ the set of system state variables, $\underline{f}$ a non-linear vector function, $\underline{h}$ the measurement function in which forward model synthetic measurements (model outputs) are generated, using the computed state variables $\underline{X}$, and $\underline{Y}$ are the measurements of interest. The state of each compartment is specified by its time-dependant dynamic pressure *P* (mmHg), inlet flow *Q* (mL/s) and volume *V* (mL):

$$\begin{array}{c} X_{k}\left(t\right)=(V_{k}\left(t\right),P_{k}\left(t\right),Q_{k}\left(t\right)), \end{array}$$ (2)

where *k* represents the compartment of interest. In generic form, the equations relating to the passive compartmental state variables all take the form:

$$\begin{array}{c} \frac{dV_{s,k}}{dt}=Q_{k}-Q_{k+1},\;\frac{dP_{k}}{dt}=\frac{1}{C_{k}}\left(Q_{k}-Q_{k+1}\right),\;Q_{k}=\frac{P_{k-1}-P_{k}}{R_{k}}. \end{array}$$ (3)

**Fig 2:**
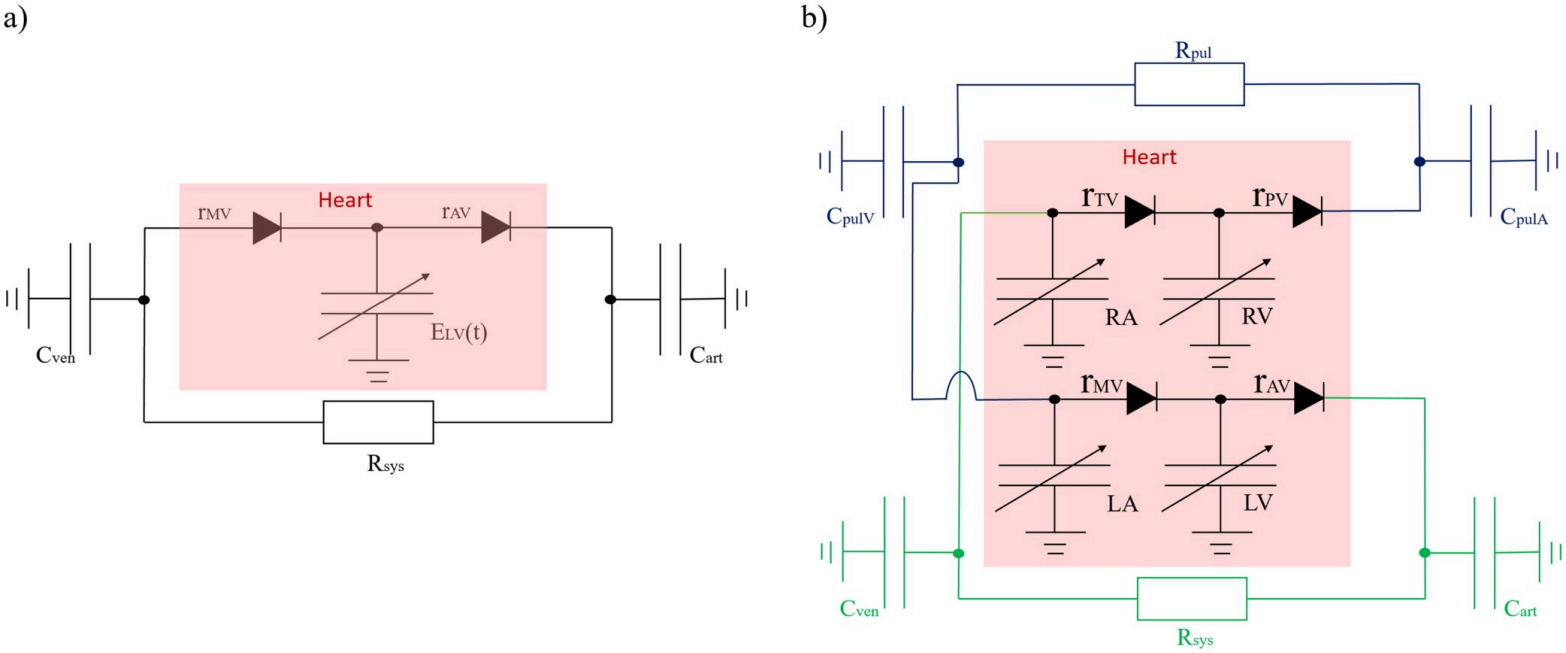
Electrical analogue representations of the one-(A) and four-chamber (B) cardiovascular models used in this work. In both, the passive circulations are represented by CRC Windkessel’s and the valves by diodes with Ohmic behaviour under both forward and reverse bias. Base parameter values (*R*_*sys*_, *C*_*art*_ etc.) are declared in Table 1. Ventricular elastance function parameters and, systemic resistance and venous compliance unstressed volumes are regulated, making it convenient to consider certain mechanical model input parameters as time-dependant states with dynamics dictated by the regulation.

Proceeding streamwise in Fig 2, the subscripts above represent the proximal (*k* − 1)^*th*^, present *k*_*th*_ and distal (*k* + 1)^*th*^ system compartments, *V*_*s*,*k*_ (mL) denotes the circulating (stressed) volume [35] and *C*_*k*_ (ml/mmHg) and *R*_*k*_ (mmHgs/mL) denote compartmental compliance and the resistance between compartments *k*, (*k* + 1). We return to the matter of unstressed volume shortly. All heart valves in this work are diodes, with small (large) resistance under forward (reverse) bias.

$$\begin{array}{c} Q_{k}=\{\begin{array}{cc} \frac{P_{k-1}-P_{k}}{r_{val}}, & P_{k-1}>P_{k},\\ \frac{P_{k-1}-P_{k}}{1000\cdot r_{val}}, & P_{k-1}\leq P_{k}, \end{array} \end{array}$$ (4)

where *r*_*val*_ represents the resistance across a heart valve.

Consider the active mechanical model compartments. The dynamics of the left ventricle (say) is described by a time-varying compliance *C*_*LV*_(*t*), or reciprocal elastance *E*_*LV*_(*t*) (mmHg/ml) [35]:

$$\begin{array}{c} E_{LV}\left(t\right)=\frac{P_{LV}\left(t\right)}{\left(V\left(t\right)-V_{0}\right)}=\frac{P_{LV}\left(t\right)}{V_{s}\left(t\right)}. \end{array}$$ (5)

Above, *V*_0_ & *V*_*s*_(*t*) represent the chamber unstressed and stressed volumes, respectively. *E*(*t*) is conveniently written as follows [36]:

$$\begin{array}{cc} E_{LV}\left(t\right) & =\left(E_{LV,max}-E_{LV,min}\right)\cdot e\left(t\right)+E_{LV,min},\\ e(t) & =\{\begin{array}{cc} \frac{1}{2}[1-\text{cos}(\frac{\pi t}{\tau_{lv,es}})], & 0\leq t<\tau_{lv,es},\\ \frac{1}{2}[1+\text{cos}(\frac{\pi (t-\tau_{lv,es})}{\tau_{lv,ep}-\tau_{lv,es}})], & \tau_{lv,es}\leq t<\tau_{lv,ep},\\ 0, & \tau_{lv,ep}\leq t<\tau , \end{array} \end{array}$$ (6)

where *e*(*t*; *τ*_*es*_, *τ*_*ep*_) is the activation function, intuitively parameterised by the end systolic and end pulse timing parameters *τ*_*es*_, *τ*_*ep*_, “contractility” *E*_*max*_, and “compliance” *E*_*min*_. Of course *τ* is the duration of the cardiac cycle and *E*_*LV*_(*t*) = *E*_*LV*_(*t* + *τ*).

#### Mechanical one-chamber model

Our one-chamber model was recently investigated by Bjordalsbakke et al., [37]. The left ventricle is represented by the Korakianitis and Shi double cosine elastance in Eq 6 and the systemic circulation by a CRC Windkessel model. Our simplest regulated system is the union of this with Ursino’s baroreflex model [10] (see section *Baroreflex Regulation*) excluding unstressed volume regulation. See Fig 2A and Tables 1 and 2. Accordingly, in our one-chamber system, regulation modulates three effectors: (i) heart period *τ*_*HR*_, (ii) maximal left ventricular elastance *E*_*LVmax*_, and (iii) systemic resistance *R*_*sys*_.

#### Mechanical four-chamber model

Our four-chamber mechanical model is declared in Fig 2B). Each chamber is represented by a Korakianitis and Shi cosine function [36]. Systemic and pulmonary circulations are both CRC Windkessels. Initial mechanical parameters for the regulated model are presented in Tables 1 and 2. In this model, five effectors were modulated to conserve arterial pressure: (i) heart period *τ*_*HR*_, (ii) maximal left ventricular elastance *E*_*LVmax*_, (iii) maximal right ventricular elastance *E*_*RVmax*_, (iv) systemic resistance *R*_*sys*_ and (v) venous unstressed volume *V*_*unV*_. See section *Unstressed Volume Regulation*.

**Table 1:** Mechanical parameters for cardiovascular one-chamber (1CH) and four-chamber (4CH) model. Adapted from [23].

Parameter	Symbol	1CH value	Source	4CH value	Source	Unit
Mean circulatory filling pressure	*mcfp*	8.00	[38]	6.00	-	*mmHg*
Heart period	*τ*_*HR*,0_	0.58	[10]	0.58	[10]	*s*
Minimal left-ventricular elastance	*E*_*LVmin*_	0.06	[39]	0.06	[39]	$\frac{mmHg}{ml}$
Maximal left-ventricular elastance	*E*_*LVmax*_	2.00	[39]	2.00	[39]	$\frac{mmHg}{ml}$
Minimal right-ventricular elastance	*E*_*RVmin*_	X	X	0.15	-	$\frac{mmHg}{ml}$
Maximal right-ventricular elastance	*E*_*RVmax*_	X	X	1.75	[10]	$\frac{mmHg}{ml}$
Initial time of ventricular systole	*τ*_*initV*_	0.00	-	0.00	-	*s*
Time of systolic phase peak—ventricles	*τ*_*S*1*V*_	0.30*τ*_*HR*_	[37]	0.30*τ*_*HR*_	[37]	*s*
Time of systolic phase end—ventricles	*τ*_*S*2*V*_	0.45*τ*_*HR*_	[40]	0.45*τ*_*HR*_	[40]	*s*
Minimal left-atrial elastance	*E*_*LAmin*_	X	X	0.15	[36]	$\frac{mmHg}{ml}$
Maximal left-atrial elastance	*E*_*LAmax*_	X	X	0.25	[36]	$\frac{mmHg}{ml}$
Minimal right-atrial elastance	*E*_*RAmin*_	X	X	0.15	[36]	$\frac{mmHg}{ml}$
Maximal right-atrial elastance	*E*_*RAmax*_	X	X	0.25	[36]	$\frac{mmHg}{ml}$
Initial time of atrial systole	*τ*_*initA*_	X	X	0.92*τ*_*HR*_	[36]	*s*
Time of systolic phase peak—atria	*τ*_*S*1*A*_	X	X	0.96*τ*_*HR*_	[36]	*s*
Time of systolic phase end—atria	*τ*_*S*2*A*_	X	X	1.0*τ*_*HR*_	-	*s*
Mitral valve resistance	*r*_*MV*_	0.06	[37]	0.0025	[10]	$\frac{mmHg\cdot s}{ml}$
Atrial valve resistance	*r*_*AV*_	0.033	[37]	0.0025	-	$\frac{mmHg\cdot s}{ml}$
Tricuspid valve resistance	*r*_*TV*_	X	X	0.0025	[10]	$\frac{mmHg\cdot s}{ml}$
Pulmonary valve resistance	*r*_*PV*_	X	X	0.0025	-	$\frac{mmHg\cdot s}{ml}$
Arterial compliance	*C*_*art*_	1.13	[37]	1.13	[37]	$\frac{ml}{mmHg}$
Systemic resistance	*R*_*sys*_	1.663	[41]	1.663	[41]	$\frac{mmHg\cdot s}{ml}$
Venous compliance	*C*_*ven*_	11.00	[37]	20.50	[36]	$\frac{ml}{mmHg}$
Pulmonary arterial compliance	*C*_*pulA*_	X	X	4.50	-	$\frac{ml}{mmHg}$
Pulmonary resistance	*R*_*pul*_	X	X	0.30	[36]	$\frac{mmHg\cdot s}{ml}$
Pulmonary venous compliance	*C*_*pulV*_	X	X	20.50	[36]	$\frac{ml}{mmHg}$
Initial venous unstressed volume	*V*_*unV*_	X	X	0.00	-	*ml*

#### Regulated and unregulated model comparison protocol

To compare sensitivities we generate an equivalent unregulated state by re-assigning input parameters in the unregulated model to the emergent values, observed in the regulated case, according to the following protocol

1. A simulation of the regulated one-chamber model is run to steady state.
2. The mean of the final 200 time points of the regulated parameter value is taken.
3. The new parameter values are utilised in the unregulated model. In the one-chamber unregulated model, the updated parameter values are *τ*_*HR*,0_ = 0.906, *E*_*LVmax*,0_ = 2.48 and *R*_*sys*,0_ = 2.386.

The parameters necessary to simulate an equivalent unregulated four-chamber model were determined from the results of regulated model execution, in the same manner as for the one-chamber model (section *Mechanical One-Chamber Model*). These values for the unregulated four-chamber model are: *R*_*sys*,0_ = 2.367, *τ*_*HR*,0_ = 0.908, *E*_*LVmax*,0_ = 2.484, *E*_*RVmax*,0_ = 2.038.

#### Baroreflex regulation

To simulate closed-loop system dynamics in response to changes in blood pressure, our mechanical and baroreflex models were coupled by the information flow and processing presented in Fig 1. See Table 2 in which Ursino’s control system parameters are defined. The baroregulation input is arterial pressure from the cardiovascular model, which is deemed a surrogate for the carotid sinus pressure *P*_*CS*_ used below: it is processed as follows after Ursino’s model [10]:

1. Arterial pressure *P*_*art*_ (our surrogate for Ursino’s carotid sinus pressure) is transduced by baroreceptors, as described in Eq 7; the solution is control pressure $\tilde{P}$, which is differenced against regulation set-point *P*_*n*_ and transformed into an afferent neural spiking frequency *f*_*CS*_ (units of spikes/s) by a sigmoidal function (Eq 8) [10].
  $$\begin{array}{c} \tau_{p}\cdot \frac{d\tilde{P}}{dt}=P_{art}+\tau_{z}\cdot \frac{dP_{art}}{dt}-\tilde{P}, \end{array}$$ (7)
  $$\begin{array}{c} f_{cs}=[f_{min}+f_{max}\cdot e^{\frac{\tilde{P}-P_{n}}{k_{a}}}]/[1+e^{\frac{\tilde{P}-P_{n}}{k_{a}}},] \end{array}$$ (8)
2. Efferent frequency is calculated for sympathetic (Eq 9: exponential) and parasympathetic (Eq 10: sigmoidal) arcs, which act simultaneously and antagonistically. Parasympathetic (vagal) activity affects only the heart period [10].
  $$\begin{array}{c} f_{es}=f_{es,\infty }+[f_{es,0}-f_{es,\infty }]\cdot e^{-k_{es}\cdot f_{cs}} \end{array}$$ (9)
  $$\begin{array}{c} f_{ev}=[f_{ev,0}+f_{ev,\infty }\cdot e^{\frac{f_{cs}-f_{cs,0}}{k_{ev}}}]/[1+e^{\frac{f_{cs}-f_{cs,0}}{k_{ev}}}] \end{array}$$ (10)
3. The efferent signals are delayed.
4. A static characteristic is calculated for the sympathetic (vagal) arcs using Eqs 11 and 12. Now, regulation action is specialised for our four- and one-chamber variants as follows. (i) four-chamber model: heart period, left ventricular elastance, right ventricular elastance, venous unstressed volume and systemic resistance are modulated. (ii) one-chamber model: heart period, left ventricular elastance and systemic resistance are modulated. The response of the effector *θ*, denoted Δ*θ*, is computed as a time series from delay differential equations (Eq 13, in which *τ*_*θ*_ is an arc-specific time constant), as discussed below. The values of regulated parameters, or effectors, are adjusted according to Eq 14, either continuously (for systemic resistance, venous unstressed volume) or at the beginning of every heart beat (heart period, heart contractility).
  $$\begin{array}{c} \sigma_{\theta ,s}\left(t\right)=\{\begin{array}{cc} G_{\theta }\cdot ln[f_{es}\left(t-D_{\theta }\right)-f_{es,min}+1] & f_{es}\geq f_{es,min}\\ 0 & f_{es}<f_{es,min} \end{array} \end{array}$$ (11)
  $$\begin{array}{c} \sigma_{T,v}\left(t\right)=G_{T,v}\cdot f_{ev}(t-D_{T,v}) \end{array}$$ (12)
  $$\begin{array}{c} \frac{d\Delta \theta }{dt}\left(t\right)=\frac{1}{\tau_{\theta }}\cdot (-\Delta \theta \left(t\right)+\sigma_{\theta }\left(t\right)) \end{array}$$ (13)
  $$\begin{array}{c} \theta \left(t\right)=\Delta \theta \left(t\right)+\theta_{0} \end{array}$$ (14)

**Table 2:** Parameters of baroreflex model. All values are taken from [10].

Parameter	Symbol	1CH value	4CH value	Unit
Regulation set-point	*P*_*n*_	92.000	92.000	*mmHg*
Minimal afferent frequency	*f*_*min*_	2.520	2.520	*spikes*/*s*
Maximal afferent frequency	*f*_*max*_	47.780	47.780	*spikes*/*s*
Slope parameter of afferent sigmoid	*k*_*a*_	11.758	11.758	*mmHg*
Central value in afferent sigmoid	*f*_*cs*,0_	25.000	25.000	*spikes*/*s*
Real pole time constant	*τ*_*p*_	2.076	2.076	*s*
Real zero time constant	*τ*_*z*_	6.370	6.370	*s*
Sympathetic frequency in infinity	*f*_*es*,∞_	2.100	2.100	*spikes*/*s*
Sympathetic frequency in zero	*f*_*es*,0_	16.110	16.110	*spikes*/*s*
Minimal sympathetic frequency	*f*_*es*,*min*_	2.660	2.660	*spikes*/*s*
Sympathetic activity coefficient	*k*_*es*_	0.0675	0.0675	*s*
Vagal frequency in zero	*f*_*ev*,0_	3.200	3.200	*spikes*/*s*
Vagal frequency in infinity	*f*_*ev*,∞_	6.300	6.300	*spikes*/*s*
Slope parameter of vagal sigmoid	*k*_*ev*_	7.060	7.060	*spikes*/*s*
Gain for *E*_*LVmax*_ regulation	*G*_*Emax*,*LV*_	0.475	0.475	$\frac{mmHg\cdot s}{ml\cdot spikes}$
Time constant for *E*_*LVmax*_ regulation	*τ*_*Emax*,*LV*_	8.000	8.000	*s*
Delay for *E*_*LVmax*_ regulation	*D*_*Emax*,*LV*_	2.000	2.000	*s*
Gain for *E*_*RVmax*_ regulation	*G*_*Emax*,*RV*_	X	0.282	$\frac{mmHg\cdot s}{ml\cdot spikes}$
Time constant for *E*_*RVmax*_ regulation	*τ*_*Emax*,*RV*_	X	8.000	*s*
Delay for *E*_*RVmax*_ regulation	*D*_*Emax*,*RV*_	X	2.000	*s*
Gain for *R*_*sys*_ regulation	*G*_*Rsys*_	0.695	0.695	$\frac{mmHg\cdot s^{2}}{ml\cdot spikes}$
Time constant for *R*_*sys*_ regulation	*τ*_*Rsys*_	6.000	6.000	*s*
Delay for *R*_*sys*_ regulation	*D*_*Rsys*_	2.000	2.000	*s*
Gain for *V*_*unV*_ regulation	*G*_*VunV*_	X	-199.000	$\frac{ml\cdot s}{spikes}$
Time constant for *V*_*unV*_ regulation	*τ*_*VunV*_	X	20.000	*s*
Delay for *V*_*unV*_ regulation	*D*_*VunV*_	X	5.000	*s*
Gain for sympathetic *τ*_*HR*_ regulation	*G*_*τ*,*s*_	-0.130	-0.130	$\frac{s^{2}}{spikes}$
Time constant for sympathetic *τ*_*HR*_ regulation	*τ*_*τ*,*s*_	2.000	2.000	*s*
Delay for sympathetic *τ*_*HR*_ regulation	*D*_*τ*,*s*_	2.000	2.000	*s*
Gain for vagal *τ*_*HR*_ regulation	*G*_*τ*,*v*_	0.090	0.090	$\frac{s^{2}}{spikes}$
Time constant for vagal *τ*_*HR*_ regulation	*τ*_*τ*,*v*_	1.500	1.500	*s*
Delay for vagal *τ*_*HR*_ regulation	*D*_*τ*,*v*_	0.200	0.200	*s*

The regulation outlined above is a faithful implementation of Ursino’s model [10]. When coupled to suitable mechanical circulation, Eqs 7 and 13 (which are responsible for effector evolution) must be solved alongside the equations describing the dynamics of mechanical model pressures. Accordingly, we treat the effectors *θ* i.e. the regulated parameters as states, *X*_*i*_(*t*) in our formulation.

### Model outputs

For the regulated one-chamber model, we perturbed 36 input parameters to examine the influence on 10 outputs. This choice was described and justified in previous work [23].

For the regulated four-chamber model, we consider the influence of the 51 model inputs on 16 mechanical model outputs: cardiac output (*CO*), heart period (*τ*_*HR*_), maximal and minimal left-ventricular volume (*V*_*LV*_), and maximal and minimal values of pressures in the following compartments; left-ventricle (*P*_*LV*_), arterial (*P*_*A*_), venous (*P*_*V*_), right-ventricle (*P*_*RV*_), pulmonary arterial (*P*_*pulA*_) and pulmonary venous (*P*_*pulV*_). Atrial pressures are given by *P*_*RA*_ = *P*_*V*_ and *P*_*LA*_ = *P*_*pulV*_. Cardiac timing parameters were excluded from analysis, due to numerical instability. The choice of the outputs emphasises pressure variations deemed suitable for future personalisation; ventricular volume was added to allow complete characterisation of one-chamber; cardiac output because it is clinically important and easily assessed and heart period because it represents the state of the regulation. In the unregulated model analysis, we excluded heart period from the outputs, because it remains constant during the whole simulation.

### Sensitivity analysis

We performed local and global sensitivity analysis for both models under both regulated and equivalent unregulated conditions.

#### Local sensitivity analysis (LSA)

Local, derivative based sensitivities are essentially forward difference approximations to partial derivatives, evaluated about a base state in input parameter space, ${\underline{\theta }}_{0}$, at time *t*. To compare influence of parameter *θ*_*i*_ evenly against the (sampled) output *X*_*j*_, we scale the raw sensitivity metric by $\frac{{\hat{\theta }}_{i}}{{\hat{y}}_{j}}$. The result is a relative sensitivity matrix $\hat{S}$ with entries ${\hat{S}}_{i,j}$. The input parameters *i* ∈ (1, …, *n*) are defined in Tables 1 and 2 and the measurements *j* ∈ (1, …, *m*) are defined in section *Model outputs*.

$$\begin{array}{c} {\hat{S}}_{i,j}\left(t\right)={\left[\frac{{\hat{\theta }}_{i}}{{\hat{y}}_{j}}\frac{\partial y_{j}\left(t\right)}{\partial \theta_{i}}\right]}_{{\underline{\theta }}_{0}}. \end{array}$$ (15)

We define relative sensitivity column vectors associated with model input *i* as follows:

$$\begin{array}{c} {\underline{\hat{S}}}_{i}={\left({\hat{S}}_{i,1},{\hat{S}}_{i,2},\ldots .,{\hat{S}}_{i,m}\right)}^{T}, \end{array}$$ (16)

where ${\hat{S}}_{i,1}$ represents the influence of the input parameter *i* against the measurement 1 (say). To compute the above sensitivity statistics, the input parameters are, of course, varied *one at a time* about ${\underline{\theta }}_{0}$.

#### Global sensitivity analysis (GSA)

Given the model of Eq 1 with *Y* a continuous or discrete output, a variance based first order or total order effect can be calculated for a generic input factor *θ*_*i*_. $\theta_{i}^{c}$ denotes a complementary set of all other model inputs, excluding *θ*_*i*_. A Sobol analysis quantifies an input parameter effect, against a specific output [42]. Both the first and total order sensitivity indices return a matrix:

$$\begin{array}{c} S=S_{i,j},\;j=1,\ldots ,m;\;i=1,\ldots ,n, \end{array}$$ (17)

where *n* and *m* represent the number of input parameters and output measurements, respectively.

The first and total order sensitivity indices can be written as:

$$\begin{array}{c} S_{1,i}\left(Y\right)=\frac{\text{Var}(\text{E}\left(Y|\theta_{i}\right))}{\text{Var}(Y)},\;S_{T,i}\left(Y\right)=\frac{\text{E}(\text{Var}\left(Y|\theta_{i}^{c}\right))}{\text{Var}(Y)}, \end{array}$$ (18)

where *S*_1,*i*_, *S*_*T*,*i*_ denote the first and total order indices’ vectors for an input parameter *θ*_*i*_ against the specific output *Y*. The sensitivity indices can be interpreted as:

$$S_{T,i}=S_{i}+\sum_{i\neq j}S_{ij}+\sum_{i\neq j\neq k}S_{ijk}+\ldots ,$$

i.e., for a given input parameter *θ*_*i*_, the total order indices are the first order indices (*θ*_*i*_’s independent effects) *plus* all higher order interactions. Thus, the difference between the total and first order can provide insight into the non-linearity associated with a model, indicating a complex response surface compared to a system which is dominated by first order effects, which would have a smoother response surface and would be simpler to personalise [43].

To quantify the convergence associated with our sensitivity indices we performed an extensive convergence investigation of a one-chamber model. To ensure convergence we used the recommended Saltelli first order estimator and the Jansen total order estimator [44, 45]. We also resampled with replacement *B* times to evaluate the certainty of the sensitivity estimate. We utilised a Quasi Monte Carlo Sobol sample to ensure adequate coverage of input space. For the convergence study we utilised the following steps for 100–2000 samples: step 100; *B* = 100; for 2000–10000 samples: step 500; *B* = 1000; for 10000–150000 samples: step 5000; *B* = 1000. We then used the results of the one- chamber convergence investigation to determine a sample size for the four-chamber model. Sobol indices require *K*(*n* + 2), where *K* is the number of samples and *n* is the number of model parameters. Table 3 compares the 4 models under investigation, their number of parameters, the number of required executions for *K* = 150*k* and the time for a model to reach a converged periodic state, from which a waveform can be extracted and metrics derived. It also underscores the need for an efficient model when performing Sobol GSA. Owing to the large number of model executions necessary, it can also be difficult to avoid exhausting memory for the accumulating solutions. Thus, high performance computing is essential. For our HPC specifications, see section *Model Implementation*.

**Table 3:** A table displaying the parameter dimension, the number of model executions and the time to reach a steady state for our 4 models’ global sensitivity analysis.

Model	1CH Reg	1CH Unreg	4CH Reg	4CH Unreg
Parameter dimension	36	9	51	22
Number of model executions	5.7 Mil	1.65 Mil	7.95 Mil	3.6 Mil
Time to steady periodic state	0.02s	0.02s	0.2s	0.09s
GSA serial execution time	31.7 Hr	8.9 Hr	441.7 Hr	90 Hr

We choose the explore ±10% from the base state of all parameters declared in Tables 1 and 2. We have chosen these limits to confine the impact of regulation in a closed-loop baro-regulated cardiovascular model to a relatively small region, centred on a physiologically plausible base state. Of course, it is the base input parameter that is perturbed for each model execution, not a regulated input parameter, or effector. The latter change, relative to the base input value, as the solution of the coupled model evolves.

### Model implementation

Our workflow utilises the Julia language [46]. Scientific computing frameworks within Julia are very efficient in solving dynamical systems compared to other popular languages [47] and their iterative solution is central to the computation of sensitivity—see section *Global Sensitivity Analysis (GSA)*. Our system was converged to a periodic state representative of rest conditions. We utilise DelayDiffEq.jl [34] with the highly efficient Tsit5 algorithm (FSAL 5th order free-interpolants method [33]), with an absolute and relative tolerance equal to 1*e*^−6^, to simulate the one- and four- chamber regulated systems. ForwardDiff.jl [48] is utilised to calculate the local sensitivities through forward mode automatic differentiation; GlobalSensitivity.jl [49] and QuasiMontecarlo.jl [50] are used to compute the global sensitivity analysis. All model code can be found in author’s Github repository. All computation was performed on the PLGrid HPC Center: ACK Cyfronet AGH on Intel(R) Xeon(R) Platinum 8268 CPU with 48 cores and 192GB RAM. A complete summary of our state space model is given in the S1 Appendix. It is important to note that were one to write the system with no state elimination, one would have to solve a delay algebraic differential equation system. To promote numerical stability of the system, we reduce it to a standard delay differential equation form, eliminating all algebraic equations. Our implementation, using Julia’s ODE solvers, as outlined above, is noteworthy in two respects, which are discussed in the remainder of this section.

#### The representation of regulated parameters, or effectors

The systemic vascular resistance (say) is, a mechanical model parameter (*R*_*sys*_) which, within a regulated system model, becomes a time-dependent effector, which take an instantaneous value based upon the sympathetic drive, the dynamics of the compartment (see Fig 1), and *R*_*sys*_. In general, mechanical effectors are *de facto* mechanical sub-model input parameters which change in time, in response to baroreflex drive. Accordingly, in the closed-loop model they are “promote” to time-dependant states, determined by certain base values (which are constant) and their particular compartmental dynamics, which of course defines the regulation. The latter must be suitably expressed. We wish to preserve both the differential formulation of the problem and the beat-to-beat regulation of heart period and contractility. To express the dynamics of these effectors in the state-space form, we note that the integral of a Dirac delta function is a Heaviside function, which may be represented as a normalised Gaussian [51] and write:

$$\begin{array}{c} \frac{d}{dt}\Delta \theta_{i}=\left(\Delta \theta_{i}-\theta_{i,prev}-\theta_{i,0}\right)\delta \left(t-t_{b}\right)=e^{-\frac{{\left(t-t_{b}\right)}^{2}}{2\sigma^{2}}}\cdot \frac{1}{\sigma }\sqrt{\frac{1}{2\pi }}\cdot \left(\Delta \theta_{i}-\theta_{i,prev}-\theta_{i,0}\right). \end{array}$$ (19)

Above, *θ*_*i*_ is a regulated parameter, *t*_*b*_ is the time of commencement of a beat, *σ* is the standard deviation of the normal distribution, Δ*θ*_*i*_(*t*) is the current value of the change in the i^th^ regulated parameter (the solution of Eq 13), Δ*θ*_*i*,*prev*_ is its value from the previous beat and *θ*_*i*,0_ is the base value. The solution of Eq 19 is the current absolute value of the parameter (state), which is preserved and can be easily accessed after computation. This approach eliminates global variables, which allows for efficient computation. With the formulation including only local variables, this allowed for easier parallelisation. For specific equations, see the S1 Appendix.

#### Unstressed volume regulation

The unstressed volume of the system venous compliance is regulated downwards as blood pressure decreases, representing an effective contraction of the vessels to restore blood pressure, following volume shifts. As an example, consider the effect of this regulation process on the dynamics of our right atrial compartment (note, that in our model *P*_*RA*_ = *P*_*V*_). For this compartment *P*_*RA*_(*t*) = *E*_*RA*_(*t*)(*V*_*RA*_(*t*) − *V*_0_). Setting the unstressed volume of the right atrium to zero, differentiating and employing a recursive substitution, we straightforwardly find:

$$\begin{array}{c} \frac{dP_{RA}}{dt}=E_{RA}i+\frac{E_{RA}^{\prime }}{E_{RA}}P_{RA}, \end{array}$$ (20)

where *i* is the net current into the right atrium, which may be determined as follows. Applying Ohm’s Law, the definition of compliance and current conservation principles anticlockwise relative to Fig 2, from the systemic arterial compartment to the right atrium, we have

$$\begin{array}{c} i=\frac{P_{A}-P_{RA}}{R_{sys}}-C_{ven}\frac{dP_{RA}}{dt}+\frac{d}{dt}\Delta \theta_{V,unstressed}-i_{TV}. \end{array}$$ (21)

Above, the terms on the right-hand side are, from left to right, the flow through the systemic resistance, the flow diverted into the venous compartment, the rate of inflow into the stressed venous compartment originating from changes in the regulated unstressed volume and finally the flow across the tricuspid valve. Substituting Eq 21 into Eq 20, we obtain the following dynamics for the stressed venous compartment:

$$\frac{dP_{RA}}{dt}=\frac{E_{RA}}{\left(1+C_{ven}E_{RA}\right)}(\frac{P_{A}-P_{RA}}{R_{sys}}+\frac{d}{dt}\Delta \theta_{V,unstressed}-i_{TV})+\frac{E_{RA}^{\prime }}{E_{RA}\left(1+C_{ven}E_{RA}\right)}P_{RA},$$

Recall, the effector for the unstressed venous volume, Δ*θ*_*V*,*unstressed*_ is a state with its own dynamics [10]. See also section *Regulated and Unregulated Model Comparison Protocol*.

## Results

### Local sensitivity analysis

**Fig 3:**
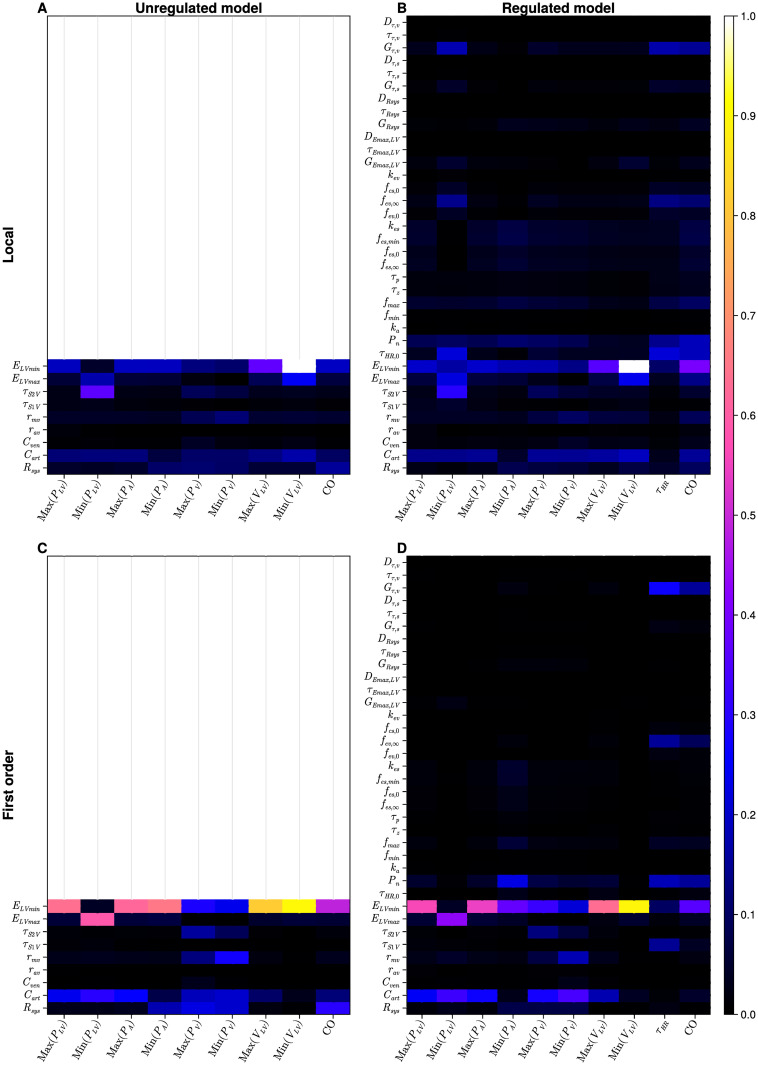
Results of a local and global sensitivity analysis of the one-chamber model. A: local analysis, unregulated model; B: local analysis, regulated model; C: global analysis, first order indices, unregulated model; D: global analysis, first order indices, regulated model. Results may be interpreted in the interval [0, 1]. Lighter colours (pink, yellow and white) identify the more influential parameters, against a specific output. Empty panels in the first column represent the unregulated models and thus no information may be compared to the regulated model in the second column.

**Fig 4:**
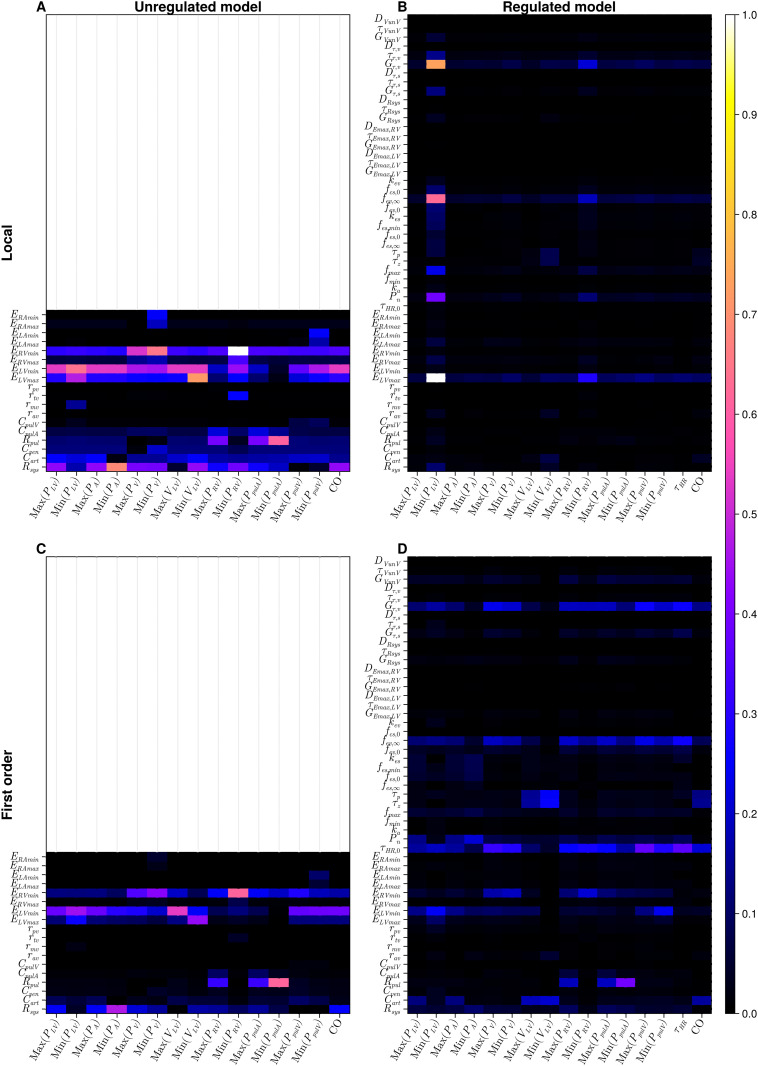
Results of global and local sensitivity analysis of the four-chamber model. A: local analysis, unregulated model; B: local analysis, regulated model; C: global analysis, first order indices, unregulated model; D: global analysis, first order indices, regulated model. Results are interpretable on the interval [0, 1]. The lighter colours (pink, yellow and white) identify the more influential parameters within the model against a specific output. The empty panels on the first column represent the unregulated models and thus only mechanical parameters are displayed compared to the regulated model in the second column.

We present results from a local relative sensitivity analysis (see Eq 15) of our one-chamber model and four-chamber model as defined in Figs 3 and 4 respectively.

#### One-chamber

For our unregulated one-chamber representation (Fig 3A), it is clear that most of the outputs are dominated by minimum elastance ($E_{LV_{min}}$), which measures the compliance of the left ventricle. The exception is minimum ventricular pressure, which is influenced principally by maximum elastance ($E_{LV_{max}}$) (contractility) and end-diastolic time (*τ*_*S*2*V*_). Results for the regulated version of the model (Fig 3B) show a pattern of mechanical parameter sensitivity which resembles the unregulated case. Parameters of the baroreflex model are generally less influential than mechanical parameters, with some exceptions among the vagal parameters, specifically the parasympathetic limit frequency (*f*_*ev*,∞_) and gain (*G*_*τ*,*v*_). For the regulation, the most consistently influential input is the set-point (*P*_*n*_).

#### Four-chamber

Results from local sensitivity analysis of the four-chamber model are presented in Fig 4C and 4D for the unregulated and the regulated systems, respectively. In the unregulated model, it is clear that systemic resistance (*R*_*sys*_) and minimum left ventricular elastance (*E*_*LVmin*_) dominate. Considering only heart parameter sensitivity, we observe that ventricular parameters dominate over atrial. Minimum and maximum atrial elastances are influential only on minimum venous pressures (both systemic and pulmonary). Notably, for heart valves, the mitral (*r*_*mv*_) and tricuspid (*r*_*tv*_) resistances affect only the minimum left ventricular pressure and right ventricular pressure respectively. Cardiac output (of high clinical significance) is influenced mostly by minimum left ventricular elastance *E*_*LVmin*_ and systemic resistance *R*_*sys*_. The sensitivity of the regulated model varies considerably relative to the results in the unregulated case. Previously dominant mechanical parameters, such as minimum left ventricular elastance *E*_*LVmin*_, minimum left ventricular elastance *E*_*RVmin*_ or systemic resistance *R*_*sys*_ do not dominate here, as in the unregulated version. The most influential input parameters are as follows: initial heart period (*τ*_*HR*,0_), baroreflex set-point (*P*_*n*_), vagal limit frequency (*f*_*ev*,∞_) and vagal gain (*G*_*τ*,*v*_). With respect to outputs, the minimum left ventricular pressure is influenced by several input parameters: overall the minimum right ventricular pressure is influenced predominantly by the most influential input parameters; the remaining outputs are less sensitive.

### Global sensitivity analysis

Here we present results from a Sobol analysis (global sensitivity analysis) of, first, the one-chamber and then the four-chamber models, for both the regulated and unregulated responses.

#### One-chamber GSA

**Fig 5:**
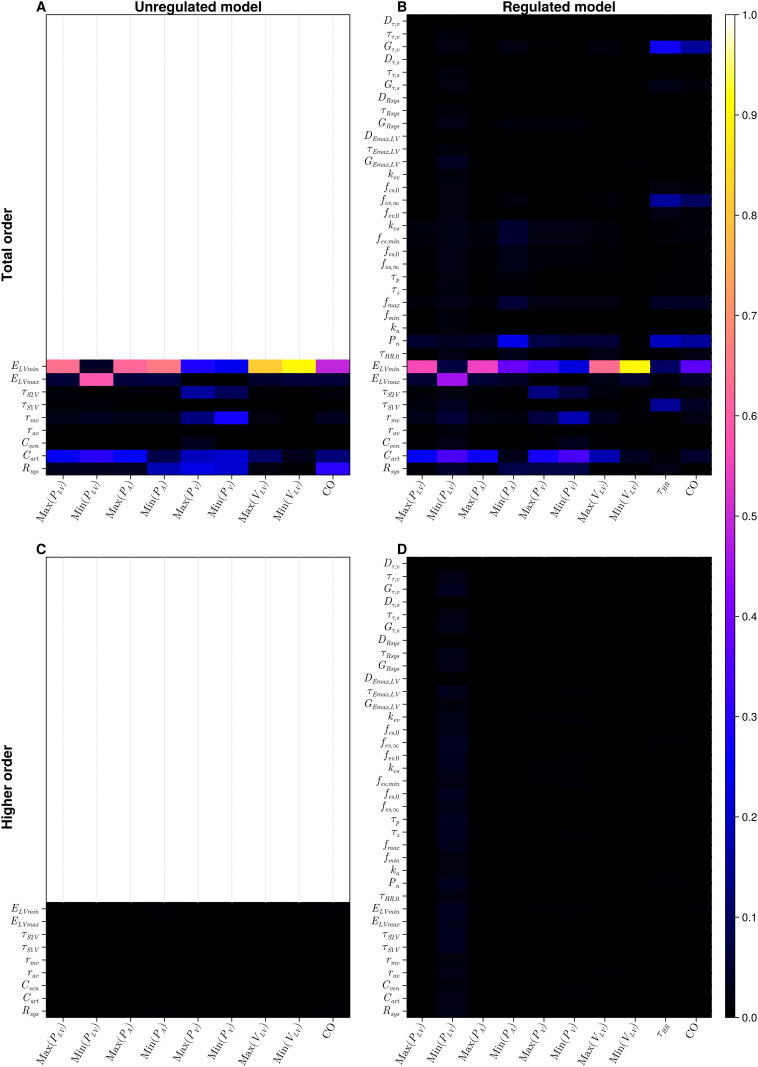
Results of global sensitivity analysis, total and higher order indices, of the one-chamber model. A: total order indices, unregulated model; B: total order indices, regulated model; C: higher order indices, unregulated model; D: higher order indices, regulated model. Results are interpretable on the interval [0, 1]. The lighter colours (pink, yellow and white) identify the more influential parameters within the model, against a specific output. The empty panels in the first column represent the unregulated models and thus no information is may be compared to the regulated model in the second column. The second row displaying the higher order indices follows by subtraction of the first order indices in the previous figure, from the total order indices above.

Comparing results of the local and global analyses Fig 3, it is clear that for the unregulated model, the sensitivity structure is very similar. For the regulated models the local and global sensitivities are noticeably different, but some trends are preserved as follows: (i) the dominance of parasympathetic parameters in determining cardiac output and heart period values; (ii) the systemic arterial compliance *C*_*art*_, minimal ventricular elastance *E*_*LVmin*_ and baroreflex set-point *P*_*n*_ are our most influential inputs; (iii) higher sensitivities are observed for mechanical parameters over regulatory parameters. Figs 3 and 5 show the results of the GSA of the one-chamber model. Apparently, there are no significant higher order interactions in the one-chamber model- see Fig 5C and 5D respectively. Global sensitivity analysis reveals that the sensitivity patterns for irst order and total order Sobol indices are almost identical, for both unregulated and regulated versions of the model. Despite these similarities, the impact of regulation of the model is still observed. In the regulated model, we observe a reduction in influence of the regulated parameters—maximum elastance (*E*_*LVmax*_) and systemic resistance (*R*_*sys*_), compared to the unregulated state. Notably, in Fig 3A, the unregulated *E*_*LVmax*_ has a first order sensitivity value of 0.59 against minimum ventricular pressure; once this same parameter is regulated, the value of the first order index is 0.45 in Fig 3B. This behaviour is also present for the systemic resistance against the maximum venous pressure, minimum venous and arterial pressure and cardiac output. Again, we underscore a persistence of patterns of sensitivity between the local and global analysis.

**Fig 6:**
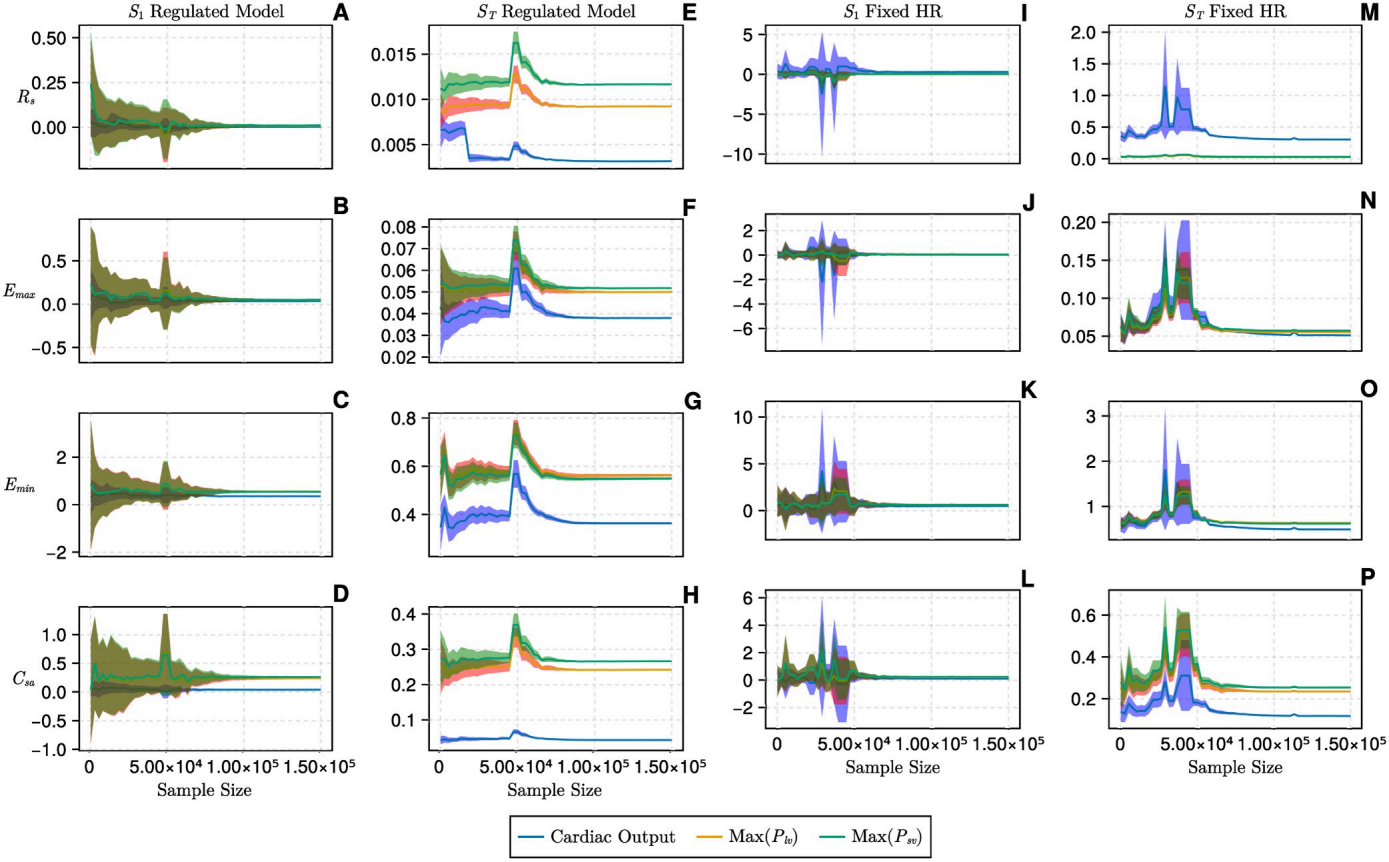
Convergence data, one-chamber model. Plots A-D show first order Sobol indices for the regulated model. Plots E-H show total order Sobol indices for the regulated model. Plots I-L show first order Sobol indices, and M-P show total order indices for the unregulated model. The parameters are displayed down the left-hand side of the first column, the parameter influence is displayed against cardiac output (blue), maximum left ventricular pressure (orange) and maximum venous pressure (green). The band around each coloured line represents the sensitivity indices 95% confidence limits of the estimated value.

We investigated the convergence behaviour of the one-chamber regulated system, compared to its unregulated version. Fig 6 shows the most influential parameters’ convergence from 100—150k samples against cardiac output, maximum ventricular pressure and maximum venous pressure outputs. The shaded area around the solid line is the 95% confidence interval. By 100k samples, both first order and total order indices have converged. In the presence of regulation, all input parameters converge with a similar behaviour as the purely mechanical unregulated model. Notably, in the presence of regulation, the first order and total order indices have smaller confidence interval limits compared to the unregulated model. The role of regulation on parameter influence can be clearly observed in panels Q and G for the cardiac output (blue). In panel Q, the unregulated systemic resistance (*R*_*sys*_) total order index value against cardiac output is around 0.4. From panel G, once regulation has been applied, the comparable sensitivity index value in the regulated state is damped below 0.005.

#### Four-chamber

Guided by one-chamber convergence data (Fig 6), we examine the results of 150k samples from our four-chamber model only—a restriction necessitated by computational cost increase factors of 4.5 and 10 for the four-chamber unregulated and regulated models, respectively. Table 4 declares the margin of error [52] which is utilised to calculate the confidence intervals associated with the sensitivity indices, at 150k samples, for all 4 of our models. For the four-chamber regulated model, although an order of magnitude more expensive than its one-chamber counterpart, indices are sufficiently small to interpret.

**Table 4:** The error associated with the sensitivity indices, measured for our 4 models. The error declared is for the maximum sample size of 150k.

Model	1CH Reg	1CH Unreg	4CH Reg	4CH Unreg
First-order mean error	0.005	0.002	0.02	0.007
First-order error range	[0.0002 − 0.02]	[0.0001 − 0.009]	[0.002 − 0.07]	[1 ⋅ 10^−4^ − 0.04]
Total-order mean error	0.0002	0.0003	0.002	0.0004
Total-order error range	[1 ⋅ 10^−8^ − 0.008]	[5 ⋅ 10^−7^ − 0.002]	[0.0006 − 0.01]	[2 ⋅ 10^−9^ − 0.007]

**Fig 7:**
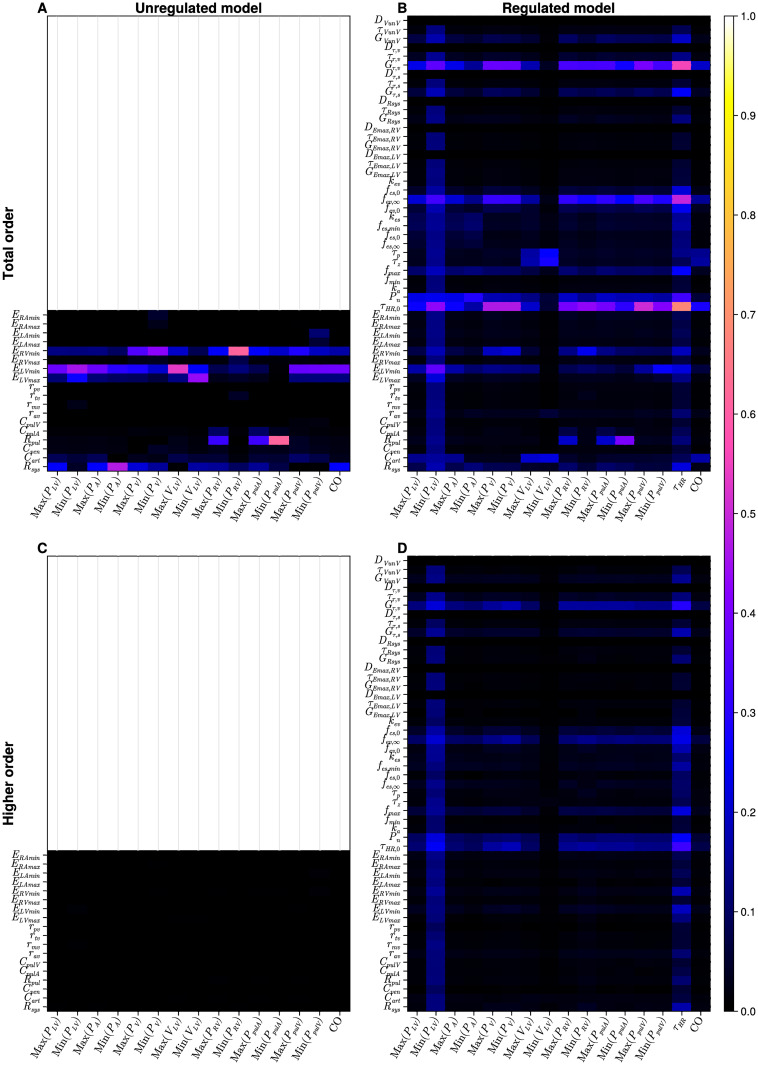
Results of global sensitivity analysis of the four-chamber model. A: total order indices, unregulated model; B: total order indices, regulated model; C: higher order indices, unregulated model; D: high order indices, regulated model. Results are interpretable on the interval [0, 1]. Lighter colours (pink, yellow and white) identify the more influential parameters within the model against a specific output. The empty panels on the first column represent the unregulated models and thus no information is displayed compared to the regulated model in the second column. The second row displaying the higher order indices is the subtraction of the first order indices in the previous figure from the total order indices above. Where there is colour, as in panel D; this indicates higher order interactions associated with a parameter.

Our results of global sensitivity analysis of the four-chamber the model are presented in Figs 4 and 7. First order Sobol indices of the unregulated four-chamber model (Fig 4C) resemble the results obtained by automatic differentiation to calculate the LSA (Fig 4A). The dominant parameters are those connected to the ventricles (except maximum right-ventricular elastance (*E*_*RVmax*_)) and systemic resistance (*R*_*sys*_). The role of minimum left atrial elastance (*E*_*LAmin*_) in determining atrial (venous) pressure *Min*(*P*_*LA*_) is clear. Panel C in Fig 7 highlights similar behaviour to the one-chamber model (Fig 5); for the unregulated model, higher order interactions are negligible. Interpreting the global sensitivities of the regulated model (panels B & D in Fig 4) is nuanced, compared to the unregulated model. We note the significance of pulmonary resistance (*R*_*pul*_) in determining pulmonary arterial pressure. We note also the influence from regulation parameters identified as sensitive in the one-chamber model (see Fig 3), specifically the regulation set point (*P*_*n*_), limit frequency (*f*_*ev*,∞_) and vagal gain (*G*_*τ*,*v*_). However, in the four-chamber model’s first and total order indices (Figs 4D and 7B), we observe more influence from other regulation parameters, than was observed in the one-chamber model. The real pole time constant *τ*_*p*_ and the real zero time constant *τ*_*z*_ are influential over the maximum and minimum ventricular volumes; we also observe increased influence of the base heart rate (*τ*_*HR*,0_) compared to the one-chamber equivalent. Panel D in Fig 7 shows how there are non-negligible higher order effects in the four-chamber regulated model, mainly for the 4 regulation parameters of base heart rate (*τ*_*HR*,0_), regulation set point (*P*_*n*_), limit frequency (*f*_*ev*,∞_) and vagal gain (*G*_*τ*,*v*_). The higher order indices exhibit a similar structure to the local indices, in Fig 4B, where minimum left ventricular elastance and regulated heart rate comprise all input parameters with non-negligible input effects.

## Discussion

We have compared the action of regulation, measured by local and global sensitivity analysis, between four heart chamber and one heart chamber 0D closed-loop models, contrasting our results to equivalent unregulated models.

Between local and global sensitivity analyses, the results from the one-chamber model showed closer agreement than the four-chamber model. The GSA also revealed that interactions between parameters for both unregulated and regulated cases were not significant for the one-chamber model. This highlights a lack of non-linearity associated with the response surface of this one-chamber model. The lack of higher order effects makes the one-chamber model a candidate for straightforward personalisation, in accord with prior art, which explores the calibration of such a model with experimental data. [43, 53].

The unregulated four-chamber model also demonstrated negligible higher order effects. However, adding regulation increased the importance of relations between input parameters, likely due to the enhanced complexity of the four-chamber model and the inclusion of volume regulation, which clearly increase the non-linearity of the response. While the physiological fidelity of the model has been increased, the difficulty attending personalisation appears to increase also. We expect high fidelity models to play a significant role in virtual representations of human physiology for e.g. digital twin applications. However, where personalisation needs to be performed at many time points, e.g. for continuous monitoring of a patient, the ease of personalisation associated with the lower fidelity one-chamber model provides a simpler alternative which may provide sufficient clinical insight.

Higher order effects were most present with vagal control of the heart period. Moreover, all analysis indicates that parasympathetic activity dominates. The sensitivity pattern achieved here is in agreement with the physiological state at which the model was simulated (all analysis in this work has been conducted at rest). Conventionally, the sensitivity results and thus the subset one obtains for personalisation is constrained by the outputs which are chosen. While this is true for the regulated class of models, we also need to consider the physiological state in which the model is operating, because a different subset of personalisable parameters may be obtained.

Comparing results from analysis of regulated and unregulated models, it is clear that the sensitivity of initial values of regulated mechanical parameters are lower than those of regulated mechanical parameters. It seems that adding a control mechanism reduces the influence of the start point, as regulation tunes the system to settle around the set-point. This explains the importance of the set-point value among baroreflex parameters, as the influence of initial values is dominated by the influence of the *P*_*n*_ value.

Another interesting observation is the highly dominant role of ventricular elastance over the atrial outputs in the four-chamber heart. This may facilitate model reduction. For example, our elastance function has 5 parameters which we show to have limited influence on the outputs, and it would be reasonable to simplify the model for the sake of a simpler parameterisation process and the elimination of sources of non-linear behaviour.

Sensitivity analysis illuminates the effects of input parameters for specific inputs. Fig 7 highlights the majority of non-linear effects are concentrated around the clinical measurement of minimum left ventricular pressure. In the process of personalisation it is vital to not only care about which parameters can be obtained from the data but which measurements one is going to utilise to calibrate the model. This vital precursor of sensitivity analysis indicates that the minimum left ventricular pressure as a measurement is influenced strongly by collective interactions of nearly all parameters in the system. Thus, any identification of parameters directly corresponding to this measurement are likely to be compromised by these non-linear effects. Such insights can be used to guide the data collected during clinical investigations to inform the personalisation process. Model personalisation typically ingests only a small amount of clinical data. Thus, given the high dimension of our input parameter spaces, it is essential to compact to a set of parameters which may be calibrated based on it. Methods such as the structural correlations method [29], or the subset selection method aim to combine the influence of the input parameters and their orthogonality [43] to address the problem. Our aim falls short of this. We present a GSA of closed-loop, coupled models, intending to determine the relative influence of rest state regulation, and to expose regulation’s impact on the sensitivity pattern. Our data correspond to a *physiological* (i.e. not pathophysiological) volume of input parameter space swept by input parameter variation ±10%, so it is appropriate to ask what is the “correct” region of input parameter space to interrogate. Exploring a larger region would certainly require an unfeasible sample number to conserve our chosen sampling density. We claim only to give here an initial examination of the impact of physiological regulation. Given that the effects of pathophysiology or different physiological states are important, a wider region of input space should eventually be targeted. Furthermore, by varying all parameters over identical relative intervals we risk over-inflating the sensitivity of certain parameters in the GSA. Compensating for this bias requires elusive prior knowledge of true physiological (let alone pathophysiological) input parameter variability- and currently baro-regulation parameters are very uncertain [10]. Nevertheless, we can clearly obtain a physiologically plausible outcomes. Further, our data—specifically, our comparison between LSA and GSA—show reassuring levels of consistency as well as intriguing differences. Perhaps our most striking observation is the dominance of vagal outflow (in agreement with Rolle et al., [17]) and a surprising lack of influence of regulation compared with the mechanical parameters overall.

### Work limitations

Baroregulation is a process which is applied across the physiological envelope. Thus, in simulating a fight-or-flight scenario (say) one may expect a different sensitivity pattern to emerge, compared with that characterising the healthy rest state, as here. In the results presented here, the sympathetic pathways show restricted influence across models. However, if one aims to simulate a different physiological state (such as body position shifts or exercise), the sensitivity pattern may well look very different (vagal influence might be expected to reduce in favour of the sympathetic). A physiological state dependence in measured sensitivities is likely to be a significant feature of regulated models, and it must complicate their personalisation.

Further, our models are predicated on heart regulation. While they are deemed adequate to expose the interactions between mechanical and regulation compartments, they have relatively low anatomical fidelity—for instance, a single compartment represents the whole systemic circulation. To obtain more detailed results, for particular parts of the cardiovascular system, a more complex, multi-compartment mechanical model must be constructed. For example, see the model of Heldt et al., [16]. Moreover, all capacitors in our models are assumed to have linear compliance, while non-linear effects may be important, especially in the presence of large changes in transmural pressure.

## Conclusion

This study presents the first global sensitivity analysis of two closed-loop, pulsatile regulated models and their unregulated counterparts, comparing trends in parameter sensitivity. An efficient state promotion method was utilised to simulate the systems, allowing for extensive and efficient analysis of parameter sensitivity convergence and influence. The results of this study form a crucial step in the further personalisation of baroregulated cardiovascular models. In this work, use of related models of differentiated complexity exposes an increasing non-linearity in model responses and the impact of this on sensitivity analysis. Including regulation decreased the direct impact of individual parameters on outputs, compared to unregulated systems. The complex four-chamber model exhibited significant higher-order sensitivities, indicating potential challenges in personalisability based on our chosen given output parameters. Both models displayed intuitive sensitivities. As they were analysed in a base state corresponding to rest, the observed dominant influence of vagal pathways on the chosen outputs was reassuring given that the baseline resting state will be maintained through vagal tone modulation, not sympathetic drive. Given that the baroreflex operates continuously, we postulate that different sensitivity results may arise during periods of increased activity (for example higher influence of sympathetic gains on the outputs), which warrants future investigation. Further work should also centre around trying to personalise these closed-loop baro-regulated cardiovascular model to aid the development of personalised health.

## Supporting information

S1 Appendix Four-chamber CRC model with regulation: Equations. (PDF)
