# Supplementary material for: Sensitivity analysis of closed-loop one-chamber and four-chamber models with baroreflex
Source: PLoS Comput Biol. 2024 Dec 23;20(12):e1012377. doi: 10.1371/journal.pcbi.1012377 (PMC11706439; doi:10.1371/journal.pcbi.1012377)
Supplement: S1 Appendix — (PDF) [file pcbi.1012377.s001.pdf]

A Four-chamber CRC model with regulation: equations

Equations (24),(31)-(36) are taken from [1]. Equations (37)-(40) are a modified version of Ursino's formulas for adjusting effector values.

Pressure in left-ventricle, based on Shi-cosine elastance function [2]:

$$\frac{dP_{LV}}{dt} = E_{localLV} \cdot (i_{MV} - i_{AV}) + \frac{\frac{dE_{localLV}}{dt} \cdot P_{LV}}{E_{localLV}} \tag{1}$$

Systemic arterial pressure:

$$\frac{dP_A}{dt} = \frac{P_A}{C_{art}R_{sys}} + \frac{P_V}{C_{art}R_{sys}} + \frac{i_{AV}}{C_{art}} \tag{2}$$

Systemic venous unstressed volume change (based on [1]):

$$\frac{d\Delta V_{unV}}{dt} = \frac{1}{\tau_{VunV}}(-\Delta V_{unV} + \sigma_{VunV}) \tag{3}$$

Right-atrial pressure, equal to systemic venous pressure, with the term describing venous unstressed volume change:

$$\frac{dP_{RA}}{dt} = -\frac{E_{localRA} \cdot \frac{d\Delta V_{unV}}{dt}}{1 + C_{ven}E_{localRA}} + \frac{\frac{dE_{localRA}}{dt} \cdot P_{RA}}{E_{localRA}(1 + C_{ven}E_{localRA})} + \frac{E_{localRA}}{1 + C_{ven}E_{localRA}} \cdot \left(\frac{P_A}{R_{sys}} - \frac{P_V}{R_{sys}} - i_{TV}\right) \tag{4}$$

$$\frac{dP_V}{dt} = \frac{dP_{RA}}{dt} \tag{5}$$

Right-ventricular pressure:

$$\frac{dP_{RV}}{dt} = E_{localRV} \cdot (i_{TV} - i_{PV}) + \frac{\frac{dE_{localRV}}{dt} \cdot P_{RV}}{E_{localRV}} \tag{6}$$

Pulmonary arterial pressure:

$$\frac{dP_{pulA}}{dt} = \frac{P_{pulA}}{C_{pulA}R_{pul}} + \frac{P_{pulV}}{C_{pulA}R_{pul}} + \frac{i_{PV}}{C_{pulA}} \tag{7}$$

Left-atrial pressure, equal to pulmonary venous pressure:

$$\frac{dP_{LA}}{dt} = \frac{\frac{dE_{localLA}}{dt} \cdot P_{LA}}{E_{localLA}(1 + C_{pulV}E_{localLA})} + \frac{E_{localLA}}{1 + C_{pulV}E_{localLA}} \cdot \left(\frac{P_{pulA}}{R_{pul}} - \frac{P_{pulV}}{R_{pul}} - i_{MV}\right) \tag{8}$$

$$\frac{dP_{pulV}}{dt} = \frac{dP_{LA}}{dt} \tag{9}$$

Control pressure $\tilde{P}$ (input for regulation):

$$\frac{d\tilde{P}}{dt} = \frac{1}{\tau_p}\left(-\tilde{P} + P_A + \tau_z \cdot \frac{dP_A}{dt}\right) \tag{10}$$

Sympathetic change of heart period:

$$\frac{d\Delta\tau_{HR,s}}{dt} = \frac{1}{\tau_{\tau,s}}(-\Delta\tau_{HR} + \sigma_{\tau,s}) \tag{11}$$

Sympathetic change of systemic resistance:

$$\frac{d\Delta R_{sys}}{dt} = \frac{1}{\tau_{Rsys}}(-\Delta R_{sys} + \sigma_{R_{sys}}) \tag{12}$$

Sympathetic change of maximum left-ventricular elastance:

$$\frac{d\Delta E_{LVmax}}{dt} = \frac{1}{\tau_{E_{LVmax}}}(-\Delta E_{LVmax} + \sigma_{E_{LVmax}}) \tag{13}$$

Sympathetic change of maximum right-ventricular elastance:

$$\frac{d\Delta E_{RVmax}}{dt} = \frac{1}{\tau_{E_{RVmax}}}(-\Delta E_{RVmax} + \sigma_{E_{RVmax}}) \tag{14}$$

Vagal change of heart period:

$$\frac{d\Delta \tau_{HR,v}}{dt} = \frac{1}{\tau_{\tau,v}}(-\Delta \tau_{HR,v} + \sigma_{\tau,v}) \tag{15}$$

Heart period τ_{HR} adjustment using Dirac delta, as described in section 2.4.1.:

$$\frac{d\tau_{HR}}{dt} = e^{-\frac{(t-t_b)^2}{2\sigma^2}} \cdot \frac{1}{\sigma}\sqrt{\frac{1}{2\pi}} \cdot (\Delta\tau_{HR,s} + \Delta\tau_{HR,s} - \tau_{HR,prev} - \tau_{HR,0}) \tag{16}$$

Maximum left-ventricular elastance E_{LVmax} adjustment using Dirac delta, as described in section 2.4.1.:

$$\frac{dE_{LVmax}}{dt} = e^{-\frac{(t-t_b)^2}{2\sigma^2}} \cdot \frac{1}{\sigma}\sqrt{\frac{1}{2\pi}} \cdot (\Delta E_{LVmax} + \Delta E_{LVmax} - E_{LVmax,prev} - E_{LVmax,0}) \tag{17}$$

Maximum right-ventricular elastance E_{RVmax} adjustment using Dirac delta, as described in section 2.4.1.:

$$\frac{dE_{RVmax}}{dt} = e^{-\frac{(t-t_b)^2}{2\sigma^2}} \cdot \frac{1}{\sigma}\sqrt{\frac{1}{2\pi}} \cdot (\Delta E_{RVmax} + \Delta E_{RVmax} - E_{RVmax,prev} - E_{RVmax,0}) \tag{18}$$

Continuous adjustment of systemic resistance R_{sys} as described in [1]:

$$R_{sys} = R_{sys,0} + \Delta R_{sys} \tag{19}$$

References

1. Ursino M. Interaction between Carotid Baroregulation and the Pulsating Heart: A Mathematical Model. The American journal of physiology. 1998 12;275:H1733-47.

2. Korakianitis T, Shi Y. Numerical simulation of cardiovascular dynamics with healthy and diseased heart valves. Journal of biomechanics. 2006;39(11):1964-82.
